# Vantage sensitivity: a framework for individual differences in response to psychological intervention

**DOI:** 10.1007/s00127-017-1471-0

**Published:** 2018-01-04

**Authors:** Bernadette de Villiers, Francesca Lionetti, Michael Pluess

**Affiliations:** 1 Department of Biological and Experimental Psychology, School of Biological and Chemical Sciences, Queen Mary University of London, G.E. Fogg Building, Office 2.01, Mile End Road, London, E1 4NS UK; 2 Centre for Economic Performance, London School of Economics, London, UK

**Keywords:** Vantage sensitivity, Environmental sensitivity, Psychiatry, Intervention, Psychotherapy

## Abstract

**Purpose:**

People differ significantly in their response to psychological intervention, with some benefitting more from treatment than others. According to the recently proposed theoretical framework of vantage sensitivity, some of this variability may be due to individual differences in environmental sensitivity, the inherent ability to register, and process external stimuli. In this paper, we apply the vantage sensitivity framework to the field of psychiatry and clinical psychology, proposing that some people are more responsive to the positive effects of psychological intervention due to heightened sensitivity.

**Methods:**

After presenting theoretical frameworks related to environmental sensitivity, we review a selection of recent studies reporting individual differences in the positive response to psychological intervention.

**Results:**

A growing number of studies report that some people benefit more from psychological intervention than others as a function of genetic, physiological, and psychological characteristics. These studies support the vantage sensitivity proposition that treatment response is influenced by factors associated with heightened sensitivity to environmental influences. More recently, studies have also shown that sensitivity can be measured with a short questionnaire which appears to predict the response to psychological intervention.

**Conclusions:**

Vantage sensitivity is a framework with significant relevance for our understanding of widely observed heterogeneity in treatment response. It suggests that variability in response to treatment is partly influenced by people’s differing capacity for environmental sensitivity, which can be measured with a short questionnaire. Application of the vantage sensitivity framework to psychiatry and clinical psychology may improve our knowledge regarding when, how, and for whom interventions work.

## Introduction

Extensive empirical evidence demonstrates that psychological intervention is an effective way to treat mental health problems [1–6]. The average beneficial effect of psychological treatment is widely accepted as substantial [7–9] and long lasting [1, 10]. However, while many people benefit from psychological therapy, between 15–45% experience no clinically significant improvement of symptoms [11–16]. This variation in treatment response is a consistent finding across all kinds of treated mental health conditions and associated therapeutic interventions. Factors well known to influence treatment response include age and sex, severity, chronicity and comorbidity of symptoms, clinician and treatment context factors, as well as social support [17–20], to mention some of the most important identified moderators of treatment response. Over the last decade, there has been pronounced scrutiny of whether the effectiveness of interventions depends also upon inherent characteristics of the individual, such as genes and personality traits. Recent psychological theories, such as vantage sensitivity, suggest that people may vary in how sensitive they are to supportive environmental influences and that this sensitivity affects their likelihood to experience the beneficial effects of psychological therapy. Hence, according to the notion of vantage sensitivity, differences in treatment outcomes may emerge—in addition to other established moderating factors—as a function of differing levels of environmental sensitivity. If such sensitivity can be measured in advance, treatment response which has direct implications for future practice may be predicted more adequately.

In this paper, we apply the concept of vantage sensitivity, a theory of how individuals respond differently to positive experiences, to the field of psychiatry and clinical psychology. We provide an up-to-date review of a selection of recent studies providing empirical evidence that genetic, physiological and psychological markers of sensitivity moderate the positive effects of psychological intervention. We then discuss the central mechanism which is hypothesized to underlie vantage sensitivity and propose existing questionnaires that can be used to measure higher-order sensitivity as a trait, before examining how increasing and converging evidence of vantage sensitivity factors may directly inform therapeutic practice.

### Theories of individual differences in environmental sensitivity

**Fig. 1:**
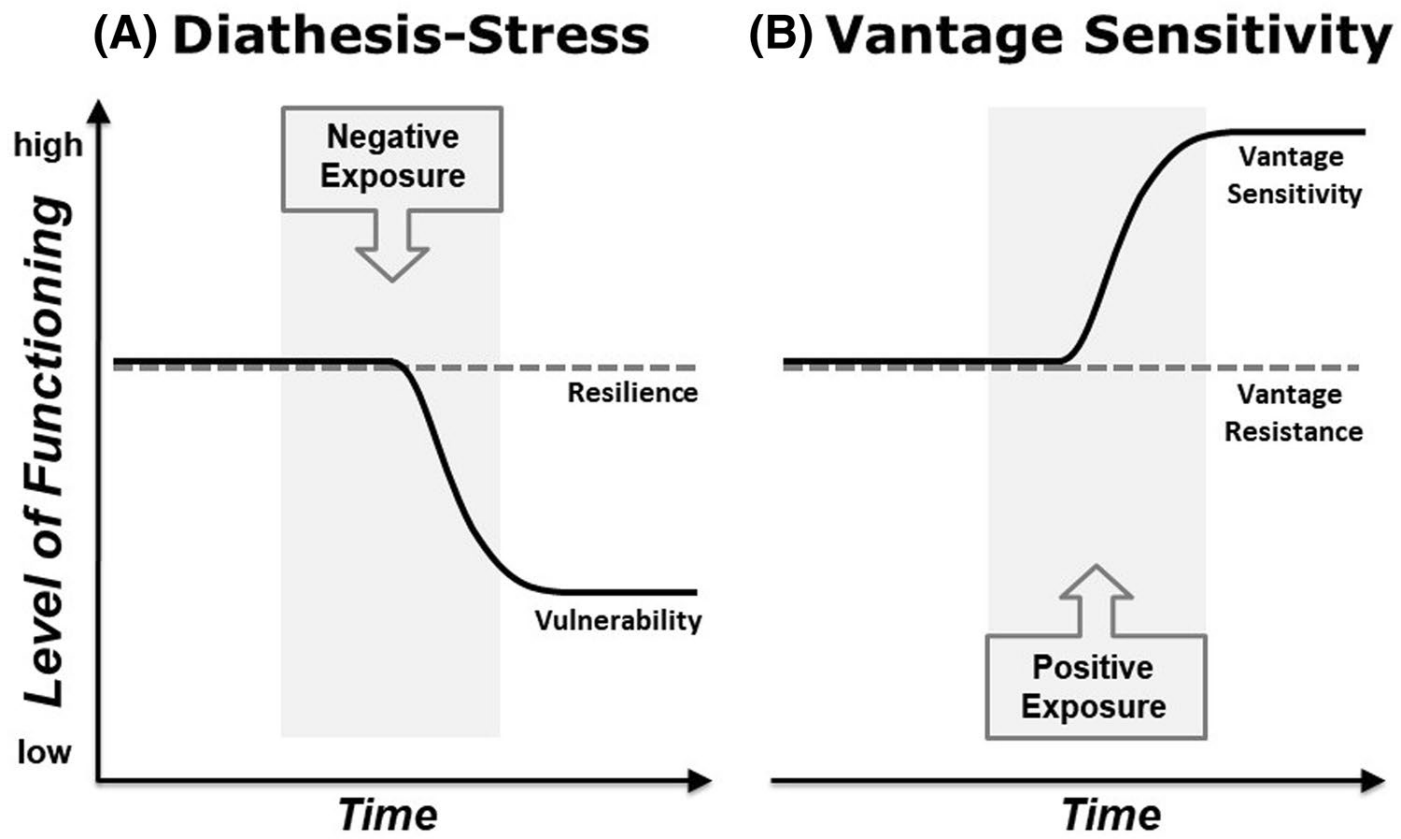
Graphic illustration of individual differences in environmental sensitivity: diathesis–stress (**a**) describes variability in response to adverse exposures, and vantage sensitivity (**b**) variability in response to supportive exposures. The combination of diathesis–stress and vantage sensitivity reflects general sensitivity to environmental influences as described by sensory processing sensitivity, differential susceptibility, and biological sensitivity to context. Adapted from “Individual Differences in Environmental Sensitivity” Fig. 1 by M. Pluess, 2015, *Child Development Perspectives* 9(3):138–143. Copyright 2015 by Wiley

The earliest systematic framework for the description of individual differences in response to environmental exposure was the diathesis–stress model. As one of the key concepts in psychopathology [21, 22], the diathesis-stress model posits that psychological problems result from the interaction between two sets of factors: (a) an individual’s inherent propensity for vulnerability, and (b) some sort of external life stressor. The model proposes that some individuals may be predisposed toward vulnerability, while others have naturally greater funds of resilience corresponding to fewer or less severe vulnerability factors, or more endogenous (e.g., genetic) resilience (see Fig. 1a).

Gene–environment interaction research, however, provides compelling evidence that the features by which we are composed do not align neatly on separate pathways to either vulnerability or resilience. Several genetic variants associated with heightened risk for maladaptive development, for example, are highly prevalent in the general population, yet in most cases do not result in maladaptive outcomes [23]. These would not be conserved should their operative properties be purely dysfunctional. Correspondingly, genetic variants found to associate with negative outcomes have been found to also associate with positive outcomes, in other circumstances [24, 25]. In more detail, numerous findings in developmental research demonstrate that the same traits that are associated with an increased risk for problematic development in negative environmental conditions also predict a higher probability to benefit from positive exposures [26–29], illustrating that vulnerability for risk and the potential for enhanced development are not mutually exclusive.

These findings converged to form an important idea, expressed in the differential susceptibility [25, 30] hypothesis: certain genetic, neurobiological, or other individual features may operate as levers, associating with different developmental outcomes depending on the quality of the environment. The theory defined by the differential susceptibility hypothesis is that some people may be more sensitive neurologically and physiologically, causing them to perceive, process, and subsequently react to experiential stimuli more strongly, both negatively and positively. This provides an explanation for the conservation of genes associated with maladaptive outcomes when experiencing adversity: the same genes may also associate with improved reproductive fitness, with the liability conferred under some conditions outweighed by advantages conferred under other conditions [31–34].

Biological sensitivity to context [35, 36] and the personality concept of sensory processing sensitivity [37] are two further important models of individual differences in sensitivity. Biological sensitivity to context demonstrates psychobiological reactivity to stress (e.g., high cortisol reactivity) with the potential to produce negative effects in adverse conditions and positive effects in supportive contexts [38, 39], whereas sensory processing sensitivity, developed by Aron (1997), is proposed as a measurable personality dimension, in which heightened sensitivity to external stimuli is attuned to greater depth of cognitive processing and high emotional reactivity [40].

Diathesis–stress, differential susceptibility, vantage sensitivity, biological sensitivity to context, and sensory processing sensitivity can all be contained under the broader conceptual meta-framework of environmental sensitivity [41]. As an overarching theoretical framework, environmental sensitivity proposes fundamental and consequential differences in how sensitively and acutely individuals perceive and absorb environmental stimuli, and that these differences—which lead some people to be more environmentally sensitive than others—are genetically influenced products of evolutionary adaptation. Importantly, the meta-framework of environmental sensitivity houses models that describe sensitivity to negative environmental exposures only, sensitivity to positive ones, as well as sensitivity to both. Although some research aims at determining statistically which of the different sensitivity models fit results of a study best [42], support for one versus other models may often depend on methodological differences between studies (e.g., type and range of outcome measures ). Hence, it may be more useful to adopt the broader view of environmental sensitivity when considering mechanisms of individual differences in response to environmental influences—whether they are negative or positive—but refer to particular models when formulating specific hypotheses or describing results. For example, in the case of individual differences in response to psychological treatment as a function of sensitivity, vantage sensitivity is the appropriate sensitivity model given that the environmental influence (i.e., psychological intervention) captures the presence/absence of a positive exposure but not the presence/absence of a negative one (which would be required to test a diathesis–stress or differential susceptibility hypothesis).

### The vantage sensitivity framework

A principal contribution of the distinct but related sensitivity models is the observation that variability in sensitivity is not confined to adversity, but operates across the full range of environmental quality. More sensitive individuals are not only more vulnerable to adversity but likely also more sensitive to the positive effect of positive experiences. Vantage sensitivity [43, 44] is a relatively new concept referring to the proclivity of some people to benefit disproportionately from positive features of environmental experience, just as vulnerability depicts a propensity to succumb to the negative effects of adversity in the diathesis–stress framework. Vantage sensitive individuals, those more responsive to and positively influenced by features of the environment that promote well-being, are presumed to possess inherent genetic, physiological and psychological traits that subserve responsivity to positive experience. Vantage-resistant individuals, on the other hand, are presumed to possess inherent characteristics that confine their likelihood of responding and altering positively to the same experiences (see Fig. 1b).

While accordant and closely related to differential susceptibility, and other models of sensitivity, vantage sensitivity is not synonymous to differential susceptibility. Given that differential susceptibility refers to individual differences in response to both adverse and positive contexts, the studied features of environmental quality must, therefore, necessarily include both negative and positive aspects to be consistent with differential susceptibility. While there is evidence that individuals highly sensitive to adverse rearing and environment factors may also be those most responsive to therapy and intervention [45], vantage sensitivity is considering individual differences that pertain only to positive experience, or the absence thereof, without making claims about the potential response to adverse experiences. In other words, vantage sensitivity describes the “bright side” of differential susceptibility, whereas diathesis–stress refers to the “dark side” only.

### Empirical evidence for vantage sensitivity

According to existing studies on individual differences in response to various positive experiences, endogenous markers of vantage sensitivity appear to fall into three different categories, as reviewed previously [43]: (a) genetic factors, such as the serotonin transporter-linked polymorphic region (5-HTTLPR) [46]; (b) physiological measures, such as cortisol reactivity [47], and (c) psychological traits, such as negative emotionality in infancy [48]. In what follows, we present a selection of new studies most of which have not been included in previous reviews [31, 43] to provide specific empirical evidence for vantage sensitivity to psychological intervention for each of the three categories.

#### Genetic factors

Several gene variants have been found to moderate the impact of psychological intervention in the so-called candidate gene–environment interaction studies [49–51]. These studies tend to focus on the moderating effect of single gene variants, such as the serotonin transporter gene polymorphism (*5-HTTLPR*) [52]. In a recent study, it was investigated whether 5-HTTLPR moderates the positive effects of a home-based intervention program aimed at promoting secure attachment in children. The randomized controlled trial included 279 South African mother–child dyads [53]. According to the results of the study, children carrying the short variant of the 5-HTTLPR were significantly more likely to be securely attached (84%) if included in the treatment condition compared to the control group (58%). In contrast, children with the long variant of the 5-HTTLPR had similar rates of secure attachment whether they were in the treatment or control group (71 and 70%, respectively). Hence, these findings suggest that the short variant of the 5-HTTLPR predicted the positive response to psychological intervention, providing evidence for vantage sensitivity as a function of 5-HTTLPR.

Similar results emerged in a randomized controlled trial aimed at investigating genetic moderation of a comprehensive intervention program focused on the prevention of externalizing behavioral problems in high-risk children [54]. The authors investigated whether several variants of the glucocorticoid receptor gene (NR3C1) influenced the response to intervention in two subsamples of European-American (*N* = 242) and African-American (*N* = 248) participants, enrolled at age 5 and tested for outcomes at age 25 years. According to the study, European-American individuals carrying the A allele of the single nucleotide polymorphism (SNP) rs10482672 had the lowest prevalence of externalizing disorders at age 25 when they belonged to the intervention group (18%), whereas those with the same gene variant in the control group had a prevalence of 75%. Conversely, for those homozygous for the G allele, no difference in externalizing symptom rates emerged between treatment (56%) and control (57%) groups. Hence, findings suggest that the A-allele reflects higher vantage sensitivity to the positive effects of psychological treatment.

Over the last years, researchers have started to combine multiple gene variants into polygenic scores based on the understanding that common and complex traits are the function of many gene variants rather than single ones [55, 56]. For example, Chhangur et al. [57] tested whether a polygenic score based on five dopaminergic gene variants (DRD4, DRD2, DAT1, MAOA, and COMT) moderated the efficacy of a parenting program applying a randomized controlled trial design involving 341 families with children characterized by elevated behavior problems. Consistent with the notion of vantage sensitivity, boys carrying 3–5 sensitivity gene variants showed the most pronounced reduction in externalizing behavioral problems both directly after the intervention and at an 8-month follow-up assessment compared to their genetically less-sensitive peers with 0–2 sensitivity variants. Importantly, at the pre-intervention assessment, children carrying more sensitivity genes did not differ from those with fewer sensitivity genes, suggesting that the polygenic score of sensitivity was unrelated to initial behavior problems.

Overcoming the limitations of candidate gene studies [58], Keers and colleagues [55] recently explored the moderating role of a genome-wide polygenic score, based on about 20,000 different gene variants, regarding the efficacy of cognitive behavioral therapy (CBT) in the treatment of 973 children with anxiety problems. Findings suggested that children with a higher genetic sensitivity score responded better to higher quality individual CBT (remission rate 70.9%) than to lower quality group or brief parent-led CBT (remission rates 55.5 and 41.6%, respectively). Conversely, no relevant differences among treatment types were identified for their genetically less sensitive peers, suggesting that more sensitive children are particularly responsive to the quality of treatment they receive.

#### Physiological factors

Several physiological characteristics have been identified as markers of sensitivity, including measures of the autonomic nervous system and hypothalamus–pituitary–adrenal (HPA) axis [36, 59, 60]. However, not many studies have yet investigated the role of these physiological characteristics from a perspective of vantage sensitivity. But evidence that cortisol reactivity functions as a marker of vantage sensitivity have been provided in a recent study on the efficacy of exposure-based psychotherapy, involving 26 female adults with panic disorder and agoraphobia [61]. Results showed that higher cortisol levels during exposure and a higher cortisol awaking response predicted faster and greater recovery. According to visual exploration of the reported interaction effects, there were no significant differences in any of the outcomes related to cortisol measures at the beginning of the intervention but over time those with higher cortisol showed more improvement than those with lower cortisol. Similar findings emerged in a recent study aimed at testing whether cortisol levels predicted the degree of reduction of post-traumatic stress disorder symptoms in a sample of 41 veterans treated with trauma-focused therapy (CBT integrated with eye movement desensitization and processing) [62]. The cortisol awakening response [2] accounted for 10% of the treatment effect, with higher CAR being associated with increased reduction of symptoms after completion of the treatment. However, a recent meta-analysis on basal cortisol as a predictor of psychological therapy response in patients with anxiety disorders [63] failed to find a significant association between cortisol levels and treatment response. This may mean that it is cortisol reactivity rather than basal levels that reflect sensitivity to the environment, a hypothesis which remains to be investigated in greater detail.

Evidence also suggests that measures of the brain, such as structure and function, could reflect individual differences in vantage sensitivity, and therefore, prove useful in the prediction of treatment response. For example, in a study involving 39 patients who underwent CBT for the treatment of social anxiety disorder, greater treatment response was predicted by greater pre-treatment brain activation in response to angry faces [64]. It was specifically regions in the dorsal and ventral occipitotemporal cortex in which initial activation for angry versus neutral faces significantly predicted treatment response. Importantly, these brain activation patterns were unrelated to initial social anxiety at pre-treatment. Further support for functional brain measures as marker of vantage sensitivity is provided in a study featuring a group of 21 patients with a diagnosis of generalized social anxiety disorder who underwent CBT [65]. Before treatment, patients participated in an attentional control task focused on emotion processing while their brain function was measured with an MRI scanner. Analyses revealed that higher activity in the right dorsal anterior cingulate cortex and less activity in the left amygdala during the pre-treatment attentional control task predicted stronger anxiety symptom improvement across the treatment.

#### Psychological factors

Personality and temperament traits have a long history in research as risk factors for the emergence of mental health problems, such as depression [66]. Over the last years, research has shown that personality and temperament traits can also reflect vantage sensitivity, by moderating intervention effects and predicting treatment response. For example, irritability in new born infants, objectively assessed within 30 days postpartum, has been shown to predict the positive effects of a parenting program in a randomized controlled trial study involving 174 mothers and their children [67]. The study showed that highly irritable children (of mothers with a secure attachment) were significantly more likely to benefit from the parenting intervention than their moderately irritable peers (i.e., 97 versus 57% probability for secure attachment, respectively). Conversely, no difference in attachment security emerged between highly and moderately irritable children in the control condition, suggesting highly irritable infants were more sensitive to the positive effects of the intervention.

Evidence of personality as moderator of intervention effects has also been reported in a recent study on a school-based intervention for children with externalizing behavioral problems [68]. Teacher-reported big five personality traits conscientiousness and extraversion emerged as significant predictors of short- and long-term treatment effects in a sample of 264 children aged 10 years. Children with lower scores of conscientiousness failed to respond to the intervention while children scoring low in extraversion (i.e., more introverted, shy children) benefited most. Interestingly, higher conscientiousness and lower extraversion have both been associated with sensory processing sensitivity [69, 70], suggesting that this more responsive personality profile may capture important components of environmental sensitivity. In a different randomized controlled trial involving 256 adolescents similar findings emerged [71]: higher conscientiousness and agreeableness both significantly predicted the positive response to a multimodal ambulant treatment for severe and persistently antisocial adolescents. However, these moderation effects only emerged at post-treatment and could no longer be observed at the follow-up assessment. Long-term effects of the moderating role of personality on treatment efficacy have been reported in a different randomized controlled trial study involving 159 female adolescents, and aimed at testing the impact of interpersonal psychotherapy for the prevention of excessive weight gain. Three years after the completion of the program, improvements in body mass index were found only among females with high trait anxiety, suggesting that trait anxiety may reflect a degree of vantage sensitivity [72].

## Discussion

### Mechanism of vantage sensitivity

Our selective review of recent empirical evidence for vantage sensitivity may suggest the involvement of different molecular, neurological, physiological, and psychological mechanisms. However, it is more likely that these different mechanisms all jointly orchestrate aspects of a higher-order mechanism of sensitivity. In other words, these various factors may reflect the same core sensitivity mechanism at different levels of analysis—a hypothesis which remains to be tested. As discussed in more detail elsewhere [34, 43, 73], there are several higher-order processes that involve the various detected individual sensitivity markers and may represent important candidate mechanisms underlying vantage sensitivity (or environmental sensitivity more generally): attentional processes, reward sensitivity, social sensitivity and stress responsivity [see 43]. These are each central nervous system processes, providing substantiation of the centrality of the nervous system in environmental sensitivity [40, 74]. According to this general hypothesis of “Neurosensitivity*”*, both genetic and environmental factors contribute to heightened sensitivity of the central nervous system which manifests itself both physiologically and psychologically [34, 41, 73, 75]. In summary, environmental sensitivity—defined as the ability to perceive and process information about the environment [41, 69]—may be driven primarily by a more sensitive central nervous system on which experiences register more easily and more deeply. Integral to this core mechanism of sensitivity is the quantifiable neurobiological trait of sensory processing sensitivity, held to influence the depth and degree by which sensory stimuli are processed, which we propose as a key candidate for the measurement of vantage sensitivity [40, 76].

### Measurement of vantage sensitivity

While all the reviewed genetic, physiological, and psychological traits appear to reflect sensitivity to positive features of the environment, they do not represent a direct measure of the proposed underlying higher-order sensitivity. Hence, they should be considered sensitivity markers—some more proximal than others—and as such may not be ideal or practical for the measurement of sensitivity. However, sensitivity to both negative and positive environmental influences can be measured reliably with the highly sensitive person (HSP) scale in adults [76] and the highly sensitive child (HSC) scale in children [69]. These are validated psychometric self-report questionnaires originally designed to capture sensory processing sensitivity, manifested in higher awareness of subtleties in the environment, heightened processing of sensory input, and the tendency to be more easily overwhelmed by emotionally and sensory stimulating environments, to name just a few of the items. First evidence of vantage sensitivity in relation to sensitivity measured with the HSC scale has been reported in a study examining individual differences in response to a school-based resilience-promoting program aimed at reducing depressive symptoms in adolescents [77]. The intervention proved effective in reducing depression symptoms up to the 6-month follow-up assessment (but was no longer significant at 12 months) [78]. When investigating whether HSC moderated treatment effects, it was found that children scoring low on the HSC scale failed to show any improvement at all (i.e., displaying vantage resistance) while those scoring high in sensitivity showed substantial reductions in depression symptoms all the way through to the 12-month follow-up assessment [77]. In other words, as hypothesized the HSC scale predicted individual differences in vantage sensitivity related to treatment response. These findings have recently been replicated in a large randomized control trial (*N* = 2024) testing the efficacy of a school-based anti-bullying intervention [79]. Although the intervention significantly decreased victimization and bullying across the whole sample, examination of moderation effects revealed that intervention effects were driven by children characterized by high sensitivity. Children scoring low on HSC, on the other hand, did not benefit from the intervention.

### Implications

There is now cogent evidence that environmental sensitivity factors explain individual differences in response to both adverse and supportive experiences [43, 44, 80]. In the context of psychotherapy, the converging evidence that people respond to a greater or lesser degree to enhancing, supportive experiences as a function of endogenous factors, hypothesized to be associated with higher-order sensitivity mechanisms, has important implications for clinical practice. Most importantly, differences in response to psychological treatment should not only be expected but already considered when making decisions about the provision of treatment. Given that sensitivity can be measured as a phenotype, there is a significant potential for the application of existing sensitivity measures such as the HSP [76] and HSC [69] scales as well as for the development of further screening tools for the detection of individuals more or less likely to respond to psychological intervention as a function of inherent sensitivity. The measurement of sensitivity before treatment contributes to a personalized medicine approach [81], allowing the clinician to select the treatment that is most likely to help the patient based on her/his individual degree of sensitivity. To enable such a personalized treatment approach, continued investigation and cataloging of sensitivity markers is crucial to achieve an objective, testable profile of vantage sensitivity and vantage resistance that can be incorporated into diagnostic and clinical practices. However, as environmental sensitivity is likely an outcome of many molecular and neurobiological mechanisms, it may be most promising to focus on measurable higher-order traits rather than the various underlying properties. Several studies using the HSC scale, for example, demonstrate that it may be possible to predict individuals least and most likely to respond to therapeutic intervention without collection of genetic and physiological data [77, 79].

The currently limited ability to accurately predict what effects a psychotherapeutic intervention is likely to have, and for whom, remains a significant challenge. One central factor that contributes to this challenge may be of predominately methodological nature: the focus on main effects when evaluating the efficacy of treatment often means that the consideration of individual differences is neglected. Group means provide no information on how much individuals differ in response to treatment. As a consequence, aggregating treatment outcomes can lead to considerable overestimation (in the case of vantage resistant individuals) and underestimation (in the case of vantage sensitive individual) of the effectiveness of interventions [14, 82]. Failure to investigate degrees of individual difference in response to treatment, and specifically to identify systematic heterogeneity in response to treatment, may explain, at least in part, the stark contrast in the successful development of drugs to treat physical illness and disease compared to the limited progress made in the treatment of psychiatric disorders [13].

The practical benefits of focusing interventions better are clear, and pertain not just to financial costs and provision of service on a more effective basis, but also to the consideration of patients for whom current therapeutic interventions do not work. While individuals most sensitive and responsive to environmental influence may require shorter or lower intensity programs of intervention, vantage-resistant individuals may require interventions of greater duration, intensity, simultaneous application of two or more treatment types—or it may be found that for some people for whom vantage resistance is very pronounced, it is not just a question of intensity and duration of therapeutic intervention(s), but a redefinition of therapeutic strategy. Importantly, being less sensitive to one type of treatment may not necessarily mean lacking sensitivity to all treatments. Future research will have to investigate whether treatment-specific vantage sensitivity factors exist and whether those that are resistant to intervention due to low sensitivity may require more intensive intervention approaches or rather alternative types of treatment (e.g., medication).

Implications of vantage sensitivity go beyond therapeutic intervention. Applications in educational and social care plans are also conceivable. For example, many children and young people in institutional care go through multiple failed residential placements, with the level and type of residential care ‘stepped up’ each time a placement breaks down. Measures to better assess the different levels of care that children require, at the point where children enter care, may have an enormous impact on the lives and developmental trajectories of many children.

There are two reasons children and young people may be a critical focus, should vantage sensitivity be incorporated into diagnostic and clinical practices. First, vantage sensitivity factors may build over time, according to positive exposures. That is, there is some evidence that biomarkers related to increased vantage sensitivity also predict their own increased expression during early developmental periods [83], in contexts of positive exposures, suggesting that individual propensity for vantage sensitivity may spiral upwards over time, subject to conditions. This leads to a second important question: whether vantage sensitivity itself can be directly influenced through intervention. While it is not possible to change genetic structure, it may be possible to increase responsivity to positive exposures, or anatomically affect biological substrates (i.e., brain structure and function), through interventions that specifically target higher-order characteristics of vantage sensitivity.

### Future directions

The notion of vantage sensitivity is still relatively new, and much remains to be investigated. Presiding questions include whether vantage sensitivity describes positive response to all kinds of positive experiences and exposures, or whether it is domain specific, with individuals differing in what types of positive exposures they are sensitive to. Whether vantage sensitivity can itself be influenced, and if so, during which the developmental periods have important social and psychological implications. More research on vantage sensitivity is required in relation to psychotherapy to replicate findings, to identify alternative therapeutic approaches for individuals that appear to show vantage resistance to standard treatment, and to develop a more fine-grained assessment of vantage sensitivity factors in the context of psychological therapy. Significant further investigation of specific endogenous factors and mechanistic pathways that predict differential response to interventions is also required. In particular, identifying endophenotypes and examining candidate genetic and neurophysiological markers of higher-order sensitivity traits as well as whether and how these various sensitivity markers are associated with self-reported sensitivity assessed with questionnaires. Furthermore, future research should also investigate whether there are specific vantage sensitivity factors that do not also increase vulnerability to the negative effects of adverse experiences.

It is eminently possible that the measurement of environmental sensitivity may not only contribute to the development of screening tools for tailored psychotherapeutic and other intervention programs, but also be the critical element therein. Measurement of environmental sensitivity is feasible with the HSP and HSC scales, and can be readily introduced into current research and clinical settings. Notwithstanding, while sensory processing sensitivity may capture important characteristics of environmental sensitivity in a robust way, more specific measures of environmental sensitivity are required, and HSP and HSC scales can be refined with more research to better predict treatment response.

## Conclusion

Heterogeneity of response to therapeutic interventions is a fact that is widely observable. According to the concept of vantage sensitivity people differ in their sensitivity to positive influences, including psychotherapy, as a function of inherent and relatively stable factors. Empirical studies confirm vantage sensitivity in relation to psychological intervention. Differences in vantage sensitivity, which can be gauged through questionnaires that measure environmental sensitivity, should be considered in clinical practice. Applying the framework of vantage sensitivity to the fields of psychiatric and psychological research may elucidate when, how, and for whom interventions work.
